# Vinyl Chloride: Sass et al. Respond

**Published:** 2005-10

**Authors:** Jennifer Beth Sass, Barry Castleman, David Wallinga

**Affiliations:** Health and Environment Program Natural Resources Defense Council, Washington, DC, E-mail: jsass@nrdc.org; Environmental Consultant, Garrett Park, Maryland; Institute for Agriculture and Trade Policy, Minneapolis, Minnesota

We provided documentation and extensive references to support two claims: industry urged the U.S. Environmental Protection Agency (EPA) to downplay data suggestive of cancer risks in tissues other than the liver, and the U.S. EPA reduced the cancer potency estimate of vinyl chloride in accordance with industry input. The American Chemistry Council (ACC) is a trade association representing over 150 companies that produce and use chemicals, including the Dow Chemical Company (Midland, MI), Georgia Gulf Corporation (Atlanta, GA), and Occidental Chemical Corporation (Los Angeles, CA) (ACC 2005). These three companies are also full members of the Vinyl Institute (Arlington, VA), whose stated goal is to “promote and protect the vinyl industry and the markets it serves” (Vinyl Institute 2005). Price, a lawyer with the ACC, maintains that our commentary is inaccurate because *a*) studies published since 1997 “reach the opposite conclusion”; *b*) by demonstrating industry influence on the U.S. EPA assessment of vinyl chloride we are disparaging of U.S. EPA scientists; and *c*) the pharmacokinetic (PK) model used by the U.S. EPA has been “peer-reviewed, published, and validated.”

In response to Price’s first point, recent studies confirm earlier findings instead of the opposite. At the time of the U.S. EPA assessment (U.S. EPA 2000) there were over 20 scientific articles and two independent reviews by the International Agency for Research on Cancer (IARC 1979, 1987) suggesting that vinyl chloride is a multisite carcinogen in humans and experimental animals. Recent reviews and data support the IARC conclusions. Of the references listed by Price, three are reviews without new data (Blair and Kazerouni 1997; Boffetta et al. 2003; Bosetti et al. 2003), two describe a PK model (Clewell et al. 2001; Reitz et al. 1996), and two contribute new data that neither refute previous studies nor support ACC claims (Mundt et al. 2000; Ward et al. 2001). One of these is a North American multicenter investigation, discussed in our commentary, which reported modest “excesses of brain cancer” and “cancer of connective and soft tissue” (Mundt et al. 2000). The second new study is a European multicenter investigation that is inconclusive regarding risks of nonliver cancers (Ward et al. 2001). Price also references a meta-analysis that actually reported an excess in brain cancer [standardized mortality ratio (SMR) = 1.26; 95% confidence interval (CI), 0.98–1.62) and soft-tissue sarcomas (SMR = 2.52; 95% CI, 1.56–4.07) (Boffetta et al. 2003); the authors of this meta-analysis concluded that “increased mortality from lung and brain cancers and from lymphatic and hematopoietic neoplasms cannot be excluded.” This is consistent with an Italian study that reported increased lung cancer deaths among polyvinyl chloride (PVC) baggers (RR = 3.04; 95% CI, 1.15–7.99) (Gennaro et al. 2003).

Price’s letter and the U.S. EPA assessment (U.S. EPA 2000) both reference a review article by National Cancer Institute authors (Blair and Kazerouni 1997). Blair stressed that his findings do not support disregarding possible risks of cancer outside the liver and that potent carcinogens such as vinyl chloride are unlikely to affect only one organ site (Blair A, personal communication). In overall mortality, a slightly increased rate of a common cancer such as lung cancer may lead to more deaths than a more markedly increased rate of a rare cancer such as liver angiosarcoma.

We believe that the U.S. EPA’s close relationship with industry compromises credibility. The ACC met with U.S. EPA regulators to discuss a vinyl chloride assessment at least 2 years before public notification of an assessment process. At the urging of the ACC (Price 1999), the U.S. EPA eliminated a statement that there is “suggestive epidemiological evidence that cancer of the brain, lung, and lymphopoietic system” associated with vinyl chloride exposure. The U.S. EPA also removed a 3-fold uncertainty factor that had been included to account for possible tumor induction at such sites, after an ACC letter called the factor “ill advised” (Price 1999). The result is that the U.S. EPA assessment (U.S. EPA 2000) does not adequately warn the public of the potential carcinogenicity of vinyl chloride suggested in the scientific literature, and the risk estimate is weakened 3-fold.

The PK model has not been validated, as stated by Price. The PK model developed by industry consultants and used by the U.S. EPA in its assessment has not been validated because assumptions used in the model have not been tested. Importantly, although the model is limited to liver effects only, the implicit assumption that all metabolism occurs in the liver is incorrect (IARC 1987; McFayden et al. 1998; U.S. EPA 2000).

By using a model limited to liver cancer, the U.S. EPA made a radical departure from its cancer guidelines, recommending that the cumulative risks of all tumor types be included in a cancer assessment (U.S. EPA 1999, 2005). The 1999 carcinogen guidelines under which vinyl chloride was assessed (U.S. EPA 1999) state that

In the analysis of animal bioassay data on the occurrence of multiple tumor types, the cancer potencies should be estimated for each relevant tumor type that is related to exposure, and the individual potencies should be summed for those tumors.

This inclusive approach is reconfirmed in the 2005 guidelines (U.S. EPA 2005). This protective approach was not taken by the U.S. EPA in its assessment of vinyl chloride cancer risks.
